# Qi Fu Yin ameliorates neuroinflammation through inhibiting RAGE and TLR4/NF-κB pathway in AD model rats

**DOI:** 10.18632/aging.205238

**Published:** 2023-11-22

**Authors:** Chunxiang He, Wenjing Yu, Miao Yang, Ze Li, Jingping Yu, Dayuan Zhong, Sisi Deng, Zhenyan Song, Shaowu Cheng

**Affiliations:** 1 School of Integrated Chinese and Western Medicine, Hunan University of Chinese Medicine, Changsha, Hunan 410208, China; 2 Key Laboratory of Hunan Province for Integrated Traditional Chinese and Western Medicine on Prevention and Treatment of Cardio-Cerebral Diseases, College of Integrated Traditional Chinese and Western Medicine, Hunan University of Chinese Medicine, Changsha, Hunan 410208, China; 3 Baoshan College of Traditional Chinese Medicine, Baoshan, Yunnan 678000, China; 4 Guangdong Provincial Hospital of Integrated Traditional Chinese and Western Medicine, Foshan, Guangdong 528000, China

**Keywords:** Alzheimer’s disease, traditional Chinese medicine, neuroinflammation, RAGE, TLR4/NF-κB signaling pathway

## Abstract

The purpose of this study is to investigate the therapeutic effect of Qi Fu Yin (QFY) on Alzheimer’s disease (AD) both computationally and experimentally. Network pharmacology analysis and molecular docking were conducted to identify potential targets and signaling pathways involved in QFY treating AD. Streptozotocin-induced AD rat model was used to verify important targets and predicted pathways. The components of QFY were identified using liquid chromatography-tandem mass spectrometry. The results indicate that the potential targets of QFY are highly enriched for anti-inflammatory pathways. Molecular docking analysis revealed stable structures formed between QFY’s active compounds, including stigmasterol, β-sitosterol, and isorhamnetin, and the identified targets. *In vivo*, QFY improved cognitive memory in AD rats and reduced the mRNA expression levels of toll-like receptor 4 (TLR4), the receptor for advanced glycation end products (AGER), and the inflammatory factors interleukin-1β (IL-1β) and tumor necrosis factor-α (TNF-α) in the brains of AD rats. Furthermore, QFY effectively reduced nuclear translocation of nuclear factor-kappa B (NF-κB) and inhibited NF-κB and microglia activation. In conclusion, QFY can ameliorate neuroinflammation in AD model rats, partly via the inhibition of TLR4 and RAGE/NF-κB pathway and microglia activation, thereby enhancing learning and memory in AD model rats.

## INTRODUCTION

Alzheimer’s disease (AD) is a degenerative disorder of the central nervous system that leads to a gradual decline in cognitive function and is the leading cause of dementia. It affects millions of people worldwide, with current estimates suggesting that around 50 million people are currently living with dementia. This number is projected to increase to nearly 75 million by 2030 and 132 million by 2050 [1]. AD has become the fourth-leading cause of mortality in modern society, following cardiovascular disease, cancer, and stroke. The economic burden of caring for AD patients is higher than the total cost of treating cardiovascular disease, cancer, and stroke combined [2]. Therefore, AD represents a significant and growing global health concern, with profound implications for both individuals and society.

Despite the prevalence of AD, its pathogenesis remains unclear, and few effective drugs are currently available for clinical use. The five AD drugs approved by the Food and Drug Administration (FDA) are all symptomatic treatments, and drugs that can modify the course of the disease are still in the early stages of research [3]. These drugs include Tacrine, Donepezil, Rivastigmine, Galantamine, and Memantine, among which the first four are acetylcholinesterase (AchE) inhibitors and Memantine is an N-Methyl-D-aspartic acid (NMDA) receptor antagonist. Both AchE inhibitors and NMDA receptor antagonists can improve cognitive and memory impairment but cannot prevent or delay the progression of the disease, and they have serious side effects. The FDA recently granted accelerated approval for aducanumab as a treatment for AD due to its ability to lower amyloid levels, which is believed to provide a clinical benefit. Despite this approval, there is still ongoing controversy surrounding the optimal dosing and duration of treatment for aducanumab [4]. Due to the complex pathogenesis of AD, the development of effective anti-AD drugs remains challenging, and many newly developed drugs have failed to show satisfactory treatment effects in recent years. Traditional Chinese medicine (TCM) has unique advantages in the treatment of AD. According to TCM theory, AD is a systemic disease categorized as “senile dementia”, which is closely related to dysfunctions of the brain, heart, liver, spleen, and kidney. The basic pathogenesis is insufficient brain marrow and vital activity disuse [5]. The earliest records of AD can be traced back to the pre-Qin period, with relevant records in traditional Chinese medicine works such as the Huang Di Nei Jing Su Wen, Shang Han Lun, and Correction of Errors in Medical Classics. Therefore, exploring the pathogenesis of AD and developing effective therapeutic drugs remain a focus of research efforts.

**Figure 1:**
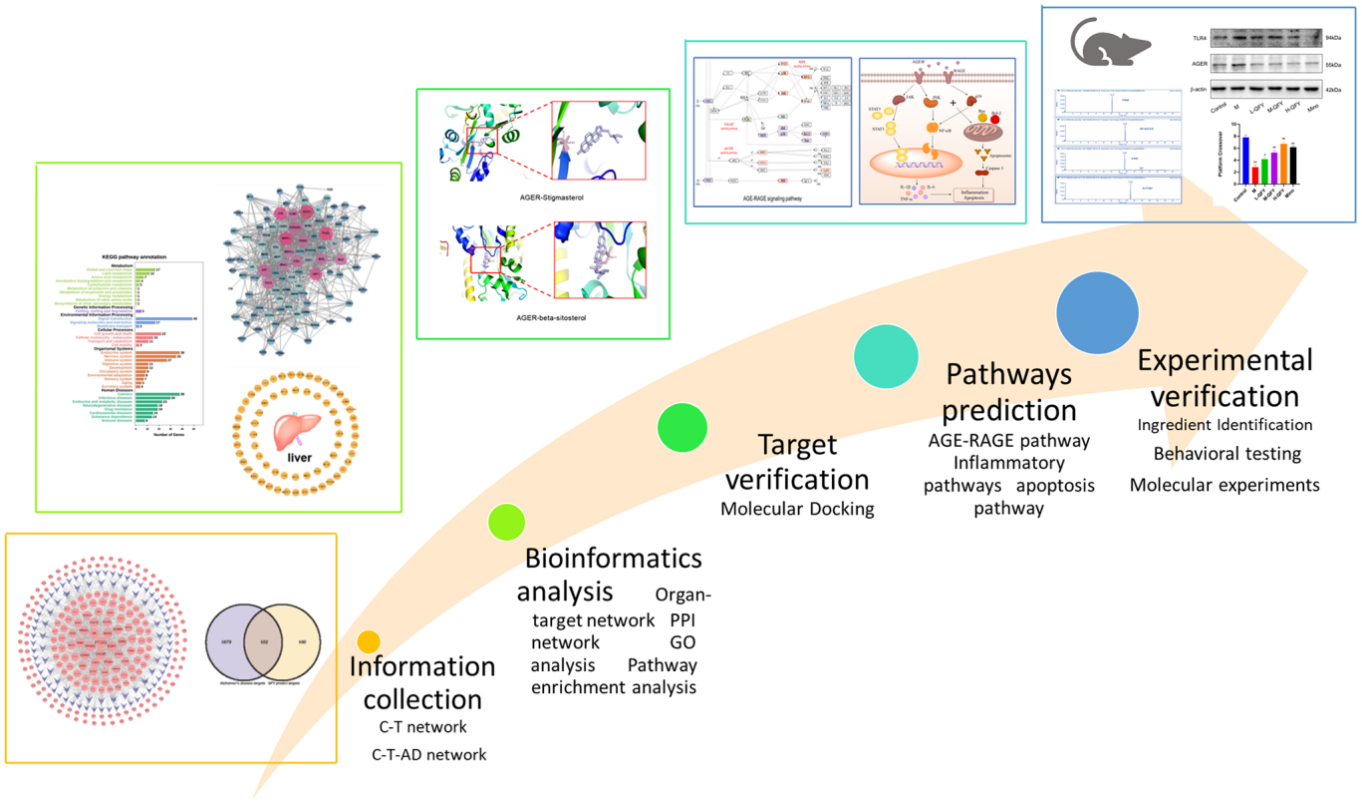
Workflow chart.

Qi Fu Yin (QFY) originated from the *Complete Works of Jingyue* written by Zhang Jiebin in the Ming Dynasty of China. The TCM prescription comprises Ginseng Radix Et Rhizoma (Renshen, RS), Rehmanniae Radix Praeparata (Shudi, SD), Angelicae Sinensis Radix (Danggui, DG), Atractylodis Macrocephalae Rhizoma (Baizhu, BZ), Polygalae Radix (Yuanzhi, YZ), Ziziphi Spinosae Semen (Suanzaoren, SZR), and Glycyrrhizae Radix Et Rhizoma Praeparata Cum Melle (Zhigancao, ZGC) (Table 1). The principles of TCM suggest that the combination of herbal ingredients in QFY can regulate vital energy, promote blood circulation, and calm the mind. Clinical studies have evaluated the efficacy of QFY in treating various types of dementia, including AD, vascular dementia (VD), and dementia syndrome [6]. Among the herbal components of QFY, angelica and ginseng have been shown to selectively inhibit the induction of inducible nitric oxide synthase (iNOS). Additionally, Rg3, an important compound found in ginseng, has potent anti-neuroinflammatory effects. The herbal formulation QFY has been shown to ameliorate neuroinflammation by downregulating iNOS in microglia [7]. Radix Rehmanniae Praeparata (Shu Dihuang, SD) is widely used as primal medicine in Chinese herbal formulas for the treatment of AD and exerts neuroprotective effects on ICV-STZ-induced AD mice through modulation of INSR/IRS-1/AKT/GSK-3β signaling pathway and intestinal microbiota [8]. Ligustilide, the main lipophilic component of Radix angelicae sinensis, has been shown to ameliorate cognitive dysfunction in a few AD mouse models. Treatment with Ligustilide ameliorates mitochondrial dynamics and morphology issues, improves mitochondrial function, reduces Aβ levels in the brain, restores the synaptic structure, and ameliorates memory deficit in APP/PS1 mice [9]. Polygalae Radix is also a traditional herbal medicine used for sedation and amnesia. Its extract prevents axonal degeneration and memory deficits by inhibiting endocytosis in a transgenic mouse model of AD [10]. Jujuboside A, a neuroprotective agent from semen Ziziphi Spinosae ameliorates behavioral disorders of the dementia mouse model induced by Aβ_1−42_ and may serve as a potential therapeutic agent for the treatment of AD [11]. Furthermore, QFY has been shown to regulate the expression of somatostatin proteins in the hippocampus of a rat model of AD, thereby improving learning and memory and potentially playing a role in AD prevention and treatment [12]. Overall, these findings offer valuable insights into the potential mechanisms through which QFY exerts its therapeutic effects on AD. They also underscore the potential of QFY as a promising and novel treatment strategy for this debilitating disease. The study used a network pharmacology approach to explore the potential mechanisms underlying the therapeutic effects of QFY in the treatment of AD. First, we retrieved and screened information on the effective compounds in QFY from the TCM database. Next, we constructed a compound-target-disease network to visualize the interrelationships among these components. Subsequently, we employed bioinformatic methods to elucidate the multitarget and multi-pathway mechanisms of QFY in controlling AD. To validate the interactions between compounds and targets predicted by network pharmacology, we conducted molecular docking experiments. Finally, we performed animal experiments to validate the results predicted in silico. A workflow chart of our study is presented in Figure 1.

**Table 1:** Component herbs of QFY.

**Chinese name**	**Latin name**	**Source species**	**Family**	**Part used**	**Place of production**	**Ingredient ratio**
Renshen	*Ginseng Radix Et Rhizoma*	*Panax ginseng* C.A.Mey.	Araliaceae	radix	Ji Lin Province, China	6
Shudi	*Radix Rehmanniae Preparata*	*Rehmannia glutinosa* (Gaertn.) DC.	Orobanchaceae	Rhizome	He Nan Province, China	9
Danggui	*Angelicae Sinensis Radix*	*Angelica sinensis* (Oliv.) Diels	Apiaceae	Radix	Gan Su Province, China	9
Baizhu	*Atractylodis Macrocephalae Rhizoma*	*Atractylodes macrocephala* Koidz.	Asteraceae	Rhizome	An Hui Province, China	5
Yuanzhi	*Polygalae Radix*	*Polygala tenuifolia* Willd.	Polygalaceae	Radix	Shan Xi Province, China	5
Suanzaoren	*Ziziphi Spinosae Semen*	*Ziziphus jujuba* Mill.	Rhamnaceae	Fructus	He Bei Province, China	6
Zhigancao	*Glycyrrhizae Radix Et Rhizoma Praeparata Cum Melle*	*Glycyrrhiza uralensis* Fisch. ex DC.	Fabaceae	Rhizome	Nei Meng Province, China	3

## MATERIALS AND METHODS

### Data preparation

The active compounds of QFY were obtained from the TCMSP [13] and BATMAN-TCM [14]. We conducted prediction and analysis of the pharmacokinetic information of the drugs to screen for active compounds. The screening conditions were set as follows: oral bioavailability (OB) ≥30%, drug-likeness (DL) ≥0.18, and blood-brain barrier (BBB) ≥−0.3. The selection of these specific parameters was based on previous studies [15].

### Target prediction

The putative targets of active compounds in QFY were obtained from the TCMSP. The SEA [16] and Binding Database [17] were used to determine the chemical similarities of the targets. Finally, all targets were standardized in UniProt [18].

### Network diagrams

To visualize the relationships between the compounds and targets in QFY, we utilized Cytoscape version 3.7.1 [19]. The resulting network diagram included two main components: nodes, representing the molecules (targets and compounds), and edges, representing the interactions between these nodes. By utilizing this powerful tool, we constructed a Compound-target (C-T) network.

#### Compound-target-AD (C-T-AD) network

To identify potential targets of QFY for treating AD, we utilized DisGeNET [20]. The Ethics Committee of Hunan University of Chinese Medicine exempted the use of public database data from ethical review. We obtained AD-related targets from DisGeNET and screened for overlaps between the predicted targets of the compounds and AD-related targets using the VENNY online tool [21]. The common targets were considered potential targets of QFY for treating AD and were imported into Cytoscape to construct the C-T-AD network.

#### Target-organ location network

To construct a target-organ localization network for the predicted targets of QFY, we utilized BioGPS, a database that provides a comprehensive resource for gene and protein function [22]. We obtained the GeneAtlas U133A and gcrma datasets from BioGPS, and then calculated the average mRNA expression levels for each target gene in the organs and identified the organs or tissues with mRNA expression levels above the average as the target organ or tissue of the gene. Using this approach, we identified the heart, liver, kidney, and brain as the main localization organs for QFY targets. Finally, we constructed a target-organ localization network using Cytoscape.

### Enrichment analysis

To gain insights into the potential biological functions and main pathways of the predicted targets of QFY for treating AD, GO and KEGG pathway enrichment analyses were carried out. Only functional terms and pathways with *q*-value < 0.05 were considered statistically significant and retained.

### Protein-protein interaction (PPI) network analysis

To gain insights into the potential PPI of the predicted targets of QFY for treating AD, we utilized the STRING online database [23]. Using a screening threshold score of 0.9, we constructed the PPI network using Cytoscape 3.7.1. Further, we carried out a Cluster module analysis to identify the potential cluster modules and gain insights into the molecular mechanisms underlying its therapeutic effects by using the Cytoscape MCODE plug-in.

### Molecular docking

The three-dimensional structures of the top target protein were obtained from the PDB [24] in pdb format. The two-dimensional structures of the compounds were downloaded from PubChem [25] as SDF files and converted to mol2 format files by Openbabel 2.4.1 [26]. Then, Molecular docking using LeDock software [27]. The lower the score, the greater the stability of the ligand and receptor. Finally, we selected the compound with the lowest docking energy for visualization, and PyMOL [28] was used to visualize the docking results.

### Animals

A total of 60 Sprague Dawley (SD) rats (150 ± 20 g) were supplied by the animal center of Hunan University of Chinese Medicine (Grade SPF, SCXK (Xiang) 2019-0004). All animal experiments were approved by the Ethics Committee of Hunan University of Chinese Medicine (No. LL-2020071501). Rats were housed in an environmentally controlled room in the SPF Laboratory Animal Center at Hunan University of Chinese Medicine, maintained on a 12-hour light/dark cycle, and provided with standard laboratory chow and water.

### Drug preparation and identification

QFY medicinal materials, including RS (SL21121302, produced in Jilin), SD (22042541C, produced in Henan), DG (TH22052407, produced in Gansu), BZ (TH220524406, produced in Anhui), YZ (2203031, produced in Shanxi), SZR (NG22052302, produced in Hebei) and ZGC (220301, produced in Neimeng). All herbs were purchased from the Pharmacy Department of the First Affiliated Hospital of Hunan University of Traditional Chinese Medicine.

As previously mentioned, QFY was prepared using both water and alcohol extraction to ensure the quality of the key components. The medicinal ingredients were combined and ground into a powder, which was then steeped for 1.5 hours in an 8-fold (v/w) 70% alcohol solution. The mixture was subjected to boiling for 0.5 hours and then simmered for an additional hour. The filtrate obtained after this process was collected. To extract any remaining components, the drug residue was subjected to another extraction process using water that had been saturated with 6-fold (v/w) 70% alcohol, following the same method. Concentrate the extract twice using a rotary evaporator. The drug residue was then extracted again using the same technique, first with 8-fold (v/w) and then with 6-fold (v/w) distilled water. The resulting water extract was added to the concentrated alcohol extract, which was heated until there was no longer any alcohol odor. The extract was then vacuum freeze-dried in a lyophilizer for two to three days, resulting in a freeze-dried QFY powder. From 100 g of raw materials, 48.8 g of lyophilized powder was obtained. Ginsenoside Rb1, atractylenolide I, rehmannioside D, ferulic acid, jujuboside A, tenuifolin, liquiritin and glycyrrhizic acid were utilized as standards for identifying the primary components of QFY to ensure quality control. In this experiment, the target compounds were separated by chromatography using a Sciex Qtrap6500+ triple quadrupole mass spectrometer liquid chromatograph on an Agilent Poroshell 120 EC-C18 4.6 × 150 mm, 4 μm liquid chromatography column. Mobile phase A was 5 mM ammonium acetate 0.1% formic acid solution, and phase B was 0.1% methanol formic acid. The column temperature was 30°C, the injection volume was 5 μl, and the flow rate was 0.8 ml/min. The gradient elution was 0–5.0 min, 95% A: 5% B; 5.0–15.0 min, 95% A: 5% B; 15.0–25 min, 5% A: 95% B; 25–25.1 min, 5% A: 95% B; 25.1–30 min, 95% A: 5% B. The mass spectrometry conditions were as follows: ion source parameters: ion source ESI; MRM mode, MRM parameters are shown in Table 2; spray voltage 5500 V; ion source temperature 550°C; curtain gas 35 psi; ion source gas 1 50 psi; ion source gas 2 50 psi; residence time 30 msec.

**Table 2:** MRM parameters table.

**Compounds**	**Polarity**	**Parent ion**	**Daughter ion**	**CE (V)**	**DP (V)**
Rehmannioside D	neg	685.1	262.9^*^	−27	−124
		685.1	179.2	−33	−124
		685.1	221.3	−34	−124
Liquiritin	neg	416.8	255^*^	−26	−64
		416.8	135	−40	−64
Jujuboside A	pos	1229.5	1229.5^*^	5	266
		1229.5	757.4	98	266
		1229.5	1083.6	99	266
		1229.5	497.1	112	266
Atractylenolide I	pos	231.3	185.1^*^	26	111
		231.3	157	31	111
		231.3	143.2	33	111
		231.3	129.3	39	111
Ginsenoside Rb1	neg	1107.4	945.8^*^	−60	−256
		1107.4	221.3	−67	−256
		1107.4	621.3	−70	−256
		1107.4	783.1	−71	−256
Tenuifolin	neg	679.1	455.4^*^	−36	−121
		679.1	425.4	−52	−121
		679.1	178.9	−36	−121
		679.1	119.1	−37	−121
		679.1	649.3	−43	−121
Ferulic Acid	neg	192.9	133.9^*^	−20	−30
		192.9	178	−17	−30
		192.9	149	−14	−30
		192.9	117.2	−21	−30
Glycyrrhizic acid	neg	821.1	821.1^*^	−5	−68

^*^Quantitative ions.

### Animal model

For this study, a concentration of 30 g/l Streptozotocin (STZ) (S0130, Sigma) powder was diluted in 0.9% sterile saline. Before treatment, the rats were fasted for 12 hours and given 0.3% pentobarbital sodium as an anesthetic. The stereotactic coordinates for intracerebroventricular (ICV) injection were as follows: dorsal-ventral (DV), 3.7 mm from the skull surface; medial-lateral (ML), 1.5 mm from the midline; and anterior-posterior (AP), 0.8 mm from the bregma. The needle was left in place for 5 minutes after bilaterally injecting STZ (2.4 mg/kg) at a rate of 0.5μl/min. After the needle was removed, the wound was treated with iodine tincture and surgically stitched up. The sham surgery group received equivalent doses of physiological saline via ICV injection. The animals were monitored for 24 hours and kept warm until they regained consciousness [29].

### Animal grouping and treatment administration

60 rats were divided into six groups using computerized randomization: sham operation, model, low-dose QFY (L-QFY, 5 g·kg−1 d), medium-dose QFY (M-QFY, 10 g·kg−1 d), high-dose QFY (H-QFY, 20 g·kg−1 d), and minocycline group (Mino, 36 mg·kg−1d). The dosages were established based on previous research and a translation of the human body surface area to that of rats. Gavage was performed a couple of days after modeling and provided twice daily for 14 days at 9:00 a.m. and 6:00 p.m., following the aforementioned dosages.

### Morris water maze (MWM)

For the local navigation test, the water’s surface was maintained at 1.5 cm below the 14 cm diameter platform, and the water temperature was kept between 23 and 25°C throughout the experiment. Each animal was given four trials per day for a total of five days. The escape time was recorded as soon as the mouse found the platform or when 60 seconds had elapsed. If the rats failed to find the platform within 60 seconds, they were guided to the platform for 10 s to encode extra maze cues. After each trial, the rats were placed back into a resting cage with a heating pad for 25 seconds before the subsequent experiment. For the spatial exploration experiment, the platform was removed, and the rats were allowed to swim freely for 60 seconds. The Morris water maze video analysis system (ZS-Morris, Beijing Zhongshi Dichuang Technology Co., Ltd.,) was utilized to record the swimming pattern of each mouse.

### Immunofluorescence

After sectioned, dried, and deparaffinized, the tissues were incubated overnight at 4°C with anti-NF-κB (bs-0465R, Bioss) and anti-Iba1(011-27991, Wako) primary antibodies. The following day, the samples were washed with PBS and incubated with fluorescent secondary antibodies (A21206; A10040, Invitrogen) at room temperature for 2 h. Finally, the samples were mounted with Antifade Solution with DAPI (S35942, Invitrogen) and kept away from light. The TissueFAXS Plus Panoramic Tissue Scanning Imaging System (Tissue Gnostics GmbH, Austria) was used to capture immunofluorescence images. Using appropriate laser excitation, representative images were selected.

### RT-qPCR

The total RNA of the hippocampus was extracted using TRIzol LS reagent (Invitrogen, USA). The reverse transcription kit (E047-01B, Novoprotein) was used for reverse transcription. Primers are shown in Table 3. The PCR reactions were performed using a MonAmp™ ChemoHS qPCR Mix (MQ00401, Monad) in CFX96TM Real-Time System (BIO-RAD, USA). The procedure involved 95°C for 10 min, followed by 40 cycles of 95°C for 5 sec., and 58°C for 30 sec., and melting curve analysis at 65–95°C for one cycle every 0.5°C to detect the fluorescence signal. The results were calculated using the 2^−ΔΔCt^ method.

**Table 3:** Primers for RT-PCR.

**Genes**		**Primers (5′–3′)**	**Product length (bp)**
NF-κB	Forward	TGTGGTGGAGGACTTGCTGAGG	138
	Reverse	AGTGCTGCCTTGCTGTTCTTGAG	
TLR4	Forward	ACTTTATCCAGAGCCGTTGGTGTATC	97
	Reverse	TCAAGGACAATGAAGATGATGCCAGAG	
IL-1β	Forward	CGTGGGATGATGACGACCTGC	161
	Reverse	GGAGAATACCACTTGTTGGCTTAT	
TNF-α	Forward	GGTCCCAACAAGGAGGAGAAGTTC	136
	Reverse	CCGCTTGGTGGTTTGCTACGAC	
β-actin	Forward	TCAGCAAGCAGGAGTACGATG	88
	Reverse	GTGTAAAACGCAGCTCAGTAACA	

### Western blotting

The total protein of the hippocampus was extracted using the RIPA technique. SDS-PAGE was performed with 30 μg of protein per well, and the separated protein on the gel was transferred onto 0.45 μm PVDF membranes and blocked in 5% skim milk for 1 h. Antibodies against RAGE (A13264, ABclonal), TLR4(220102, Sangon), β-actin (AF7018, Affinity) and secondary antibody (AP124; AP13P, Sigma). The expression of the target protein was normalized to β-actin protein.

### Statistical analysis

Statistical analysis was performed using IBM SPSS Statistics 25.0. The measurement data were presented as the means ± standard deviation ($\bar{x}\pm s$). Two-sample *t*-tests were used to compare two groups, while one-way analysis of variance (ANOVA) was used to compare more than two groups. The memory curve of the water maze was compared using two-way ANOVA. *P* < 0.05 was considered statistically significant. GraphPad 8.0.1 was used to construct the statistical graphs.

## RESULTS

### Screening of active compounds

A total of 190 compounds were collected from RS, 76 from SD, 125 from DG, 55 from BZ, 280 from ZGC, and 33 from SZR. YZ did not have any compounds listed in the TCMSP database; however, 11 active compounds were reported in the literature [30–33]. After the screening, there were 87 active compounds of QFY remained for subsequent analysis (Table 4).

**Table 4:** Information of the Qi Fu Yin (QFY) compounds^a^.

**No.**	**Molecule name**	**OB (%)**	**BBB**	**DL**	**Herb**
C1	(2R)-7-hydroxy-2-(4-hydroxyphenyl) chroman-4-one	71.12	−0.25	0.18	*ZGC*
C2	(2S)-7-hydroxy-2-(4-hydroxyphenyl)-8-(3-methylbut-2-enyl) chroman-4-one	36.57	−0.04	0.32	*ZGC*
C3	(S)-Coclaurine	42.35	0.06	0.24	*SZR*
C4	1,7-Dimethoxyxanthone	56.87	–	0.3336	*YZ*
C5	1-Methoxyphaseollidin	69.98	0.48	0.64	*ZGC*
C6	3,4,5-Trimethoxycinnamic acid	–	–	–	*YZ*
C7	3,4-Dimethoxycinnamic acid	39.58	–	0.3994	*YZ*
C8	3′-Hydroxy-4′-O-Methylglabridin	43.71	0.73	0.57	*ZGC*
C9	3′-Methoxyglabridin	46.16	0.47	0.57	*ZGC*
C10	3β-acetoxyatractylone	54.07	1.08	0.22	*BZ*
C11	6-prenylated eriodictyol	39.22	−0.29	0.41	*ZGC*
C12	7-Acetoxy-2-methylisoflavone	38.92	0.16	0.26	*ZGC*
C13	7-Methoxy-2-methyl isoflavone	42.56	0.56	0.2	*ZGC*
C14	8β-ethoxy atractylenolide III	35.95	1.12	0.21	*BZ*
C15	Alexandrin_qt	36.91	0.88	0.75	*RS*
C16	Aposiopolamine	66.65	0.4	0.22	*RS*
C17	Arachidonate	45.57	0.58	0.2	*RS*
C18	Beta-sitosterol	36.91	0.99	0.75	*DG /RS*
C19	Daucosterol	36.91	1.15	0.75	*SZR*
C20	Dehydroglyasperins C	53.82	−0.12	0.37	*ZGC*
C21	Deoxyharringtonine	39.27	−0.25	0.81	*RS*
C22	DFV	32.76	−0.29	0.18	*ZGC*
C57	Isorhamnetin	49.6	−0.54	0.31	*ZGC*
C23	Diop	43.59	0.26	0.39	*RS*
C24	Euchrenone	30.29	0.39	0.57	*ZGC*
C25	Eurycarpin A	43.28	−0.06	0.37	*ZGC*
C26	Formononetin	69.67	0.02	0.21	*ZGC*
C27	Frutinone A	65.9	0.46	0.34	*RS*
C28	Fumarine	59.26	−0.13	0.83	*RS*
C29	Gadelaidic acid	30.7	0.94	0.2	*ZGC*
C30	Gancaonin A	51.08	0.13	0.4	*ZGC*
C31	Gancaonin B	48.79	^−^0.1	0.45	*ZGC*
C32	Gancaonin G	60.44	0.23	0.39	*ZGC*
C33	Gancaonin H	50.1	^−^0.14	0.78	*ZGC*
C34	Ginsenoside-Rh4_qt	31.11	^−^0.18	0.78	*RS*
C35	Girinimbin	61.22	1.22	0.31	*RS*
C36	Glabranin	52.9	0.31	0.31	*ZGC*
C37	Glabrene	46.27	0.04	0.44	*ZGC*
C38	Glabridin	53.25	0.36	0.47	*ZGC*
C39	Glabrone	52.51	−0.11	0.5	*ZGC*
C40	Glepidotin A	44.72	0.06	0.35	*ZGC*
C41	Glepidotin B	64.46	−0.09	0.34	*ZGC*
C42	Glyasperin B	65.22	−0.09	0.44	*ZGC*
C43	Glyasperin C	45.56	0.07	0.4	*ZGC*
C44	Glyasperin F	75.84	−0.15	0.54	*ZGC*
C45	Glyasperins M	72.67	−0.04	0.59	*ZGC*
C46	Glycyrin	52.61	−0.13	0.47	*ZGC*
C47	Glycyrol	90.78	−0.2	0.67	*ZGC*
C48	Glypallichalcone	61.6	0.23	0.19	*ZGC*
C49	Glyzaglabrin	61.07	−0.2	0.35	*ZGC*
C50	Harmine	56.8	0.79	0.13	*YZ*
C51	HMO	38.37	0.25	0.21	*ZGC*
C52	Icos-5-enoic acid	30.7	1.09	0.2	*ZGC*
C53	Inermin	65.83	0.36	0.54	*RS*
C54	Inermine	75.18	0.4	0.54	*ZGC*
C55	Inflacoumarin A	39.71	−0.24	0.33	*ZGC*
C56	Isoglycyrol	44.7	0.05	0.84	*ZGC*
C58	Isotrifoliol	31.94	−0.25	0.42	*ZGC*
C59	Jaranol	50.83	−0.22	0.29	*ZGC*
C60	Kanzonol F	32.47	0.56	0.89	*ZGC*
C61	Kanzonols W	50.48	0.04	0.52	*ZGC*
C62	Licoagrocarpin	58.81	0.61	0.58	*ZGC*
C63	Licoagroisoflavone	57.28	0.09	0.49	*ZGC*
C64	Licochalcone a	40.79	−0.21	0.29	*ZGC*
C65	Licochalcone G	49.25	−0.04	0.32	*ZGC*
C66	Licocoumarone	33.21	0.06	0.36	*ZGC*
C67	Licoisoflavanone	52.47	−0.22	0.54	*ZGC*
C68	Licoisoflavone	41.61	−0.27	0.42	*ZGC*
C69	Licoisoflavone B	38.93	−0.18	0.55	*ZGC*
C70	Licoricone	63.58	−0.14	0.47	*ZGC*
C71	Lupiwighteone	51.64	−0.23	0.37	*ZGC*
C72	Mairin	55.38	0.22	0.78	*SZR/ZGC*
C73	Medicarpin	49.22	0.53	0.34	*ZGC*
C74	Odoratin	49.95	−0.24	0.3	*ZGC*
C75	Panaxadiol	33.09	0.23	0.79	*RS*
C76	Phaseol	78.77	−0.06	0.58	*ZGC*
C77	Phaseolinisoflavan	32.01	0.46	0.45	*ZGC*
C78	Phytosterol	36.91	1.16	0.75	*SZR*
C79	Polygalacic acid	31.43	−1.46	0.7	*YZ*
C80	Sanjoinenine	67.28	−0.24	0.79	*SZR*
C81	Shinpterocarpin	80.3	0.68	0.73	*ZGC*
C82	Sitosterol	36.91	0.87	0.75	*SD/ZGC*
C83	Stigmasterol	43.83	1	0.76	*RS/SD/DG*
C84	Suchilactone	57.52	0.28	0.56	*RS*
C85	Vestitol	74.66	0.3	0.21	*ZGC*
C86	Xambioona	54.85	0.52	0.87	*ZGC*
C87	Zizyphusine	41.53	0.6	0.55	*SZR*

^a^Information of the Qi Fu Yin (QFY) compounds from the Traditional Chinese Medicine Systems Pharmacology Database and Analysis Platform (TCMSP) (https://tcmsp-e.com/tcmsp.php) selected by the screening conditions oral bioavailability (OB) ≥30%, blood-brain-barrier (BBB) ≥0.3, and drug-likeness (DL) ≥0.18. Ginseng Radix Et Rhizoma (*Panax ginseng* C.A. Mey., Renshen, RS), Rehmanniae Radix Praeparata (*Rehmannia glutinosa* (Gaertn.) DC., Shudi, SD*),* Angelicae Sinensis Radix (*Angelica sinensis* (Oliv.) Diels, Danggui, DG), Atractylodis Macrocephalae Rhizoma (*Atractylodes macrocephala* Koidz., Baizhu, BZ), Polygalae Radix (*Polygala tenuifolia* Willd., Yuanzhi, YZ*),* Ziziphi Spinosae Semen (*Ziziphus jujuba* Mill., Suanzaoren, SZR), and Glycyrrhizae Radix Et Rhizoma Praeparata Cum Melle (*Glycyrrhiza uralensis Fisch. ex DC.*, Zhigancao, ZGC).

### Targets of filtered compounds

**Figure 2:**
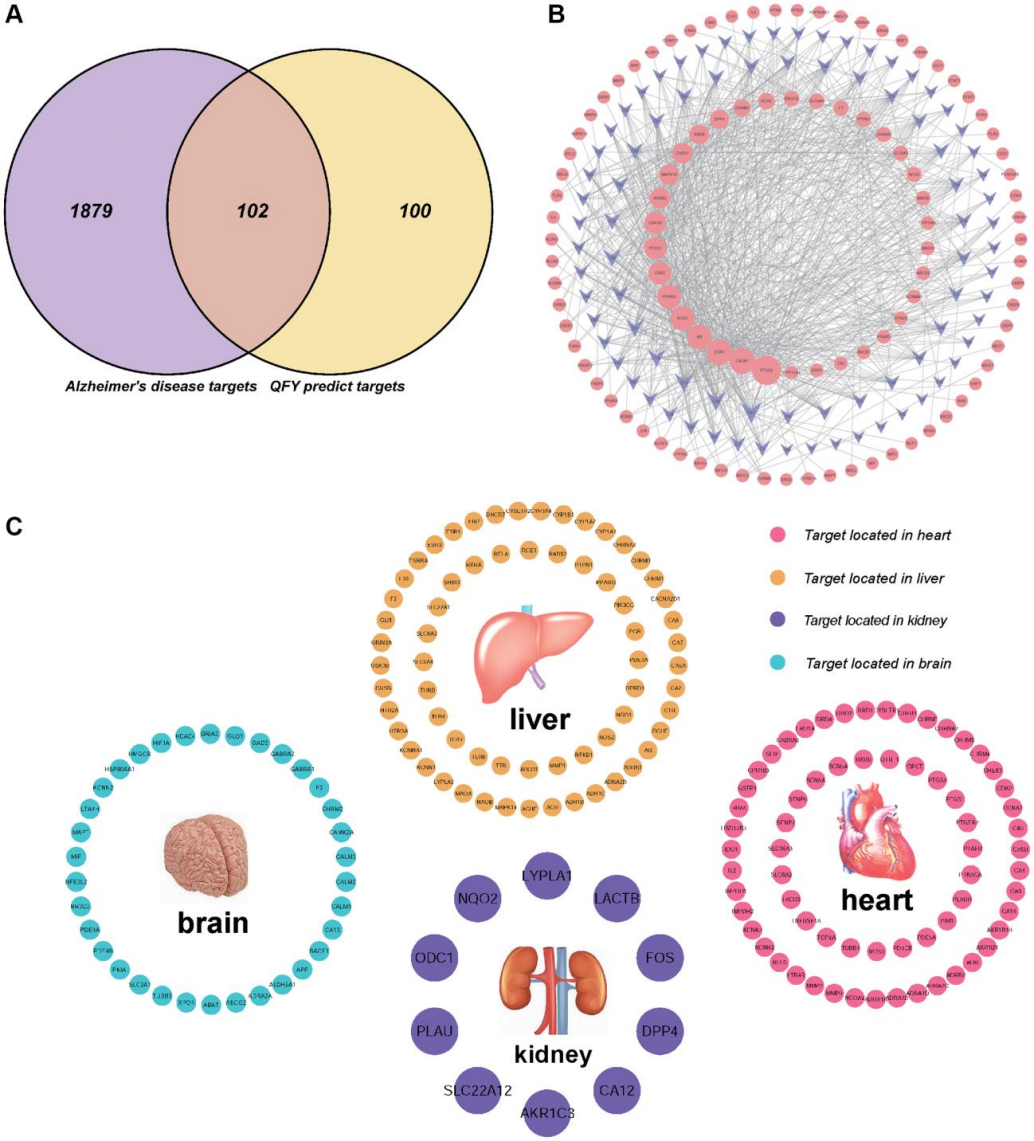
**A visualization network construction and target-organ localization network for the treatment of Alzheimer’s disease (AD) with Qi Fu Yin (QFY) established by network pharmacology.** (**A**) The targets related to AD among those predicted by QFY were selected. Purple represents Alzheimer’s disease targets, yellow represents QFY-predicted targets, and pink represents targets related to AD among those predicted by QFY. (**B**) Compound-Target-Disease (C-T-D) network: compound-target-AD network in which the purple nodes represent compounds, the pink nodes represent targets, and the lines between the nodes represent the interactions between compounds and targets. (**C**) Target-organ localization network. The screened target genes were used to establish a network with 84 human organs, including the heart (66 targets, red), liver (65 targets, yellow), kidney (10 targets, purple), and brain (34 targets, blue). The details refer to Supplementary Material 3: Supplementary Table 3.

A total of 203 predictive targets were obtained from the 87 selected active compounds through database screening, literature retrieval, and ligand structure characteristics (Supplementary Table 1). These 203 targets were mapped to the 1981 AD-related gene networks obtained from the DisGeNET database. QFY was found to be closely related to the control of AD, with 102 targets identified in this study (Figure 2A, Supplementary Table 2).

### Network relationships

#### C-T network

The interactions between the 87 selected active compounds and 203 targets in QFY were visualized by the C-T network (Supplementary Figure 1). The network contained 292 nodes and 1455 edges. Targets located in the outer circles were connected to at least two compounds, while targets in the inner circle were connected to more compounds. On average, each node was connected to 9.918 adjacent nodes. Among the 87 active compounds, 48 were found to have more than 20 functional targets, with the highest number being 69, indicating that these components may play a significant role in the therapeutic efficacy of QFY.

#### C-T-AD network

The association between 87 active compounds and 102 potential AD target genes was visualized by the C-T-AD network (Figure 2B). A higher degree indicated that the compound had more associated targets [34]. Among them, isorhamnetin (C57, degree = 67), formononetin (C26, degree = 45), 7-methoxy-2-methyl isoflavone (C13, degree =42), and β-sitosterol (C18, degree = 40) had the highest degrees and were located at the center of the network. Meanwhile, 17 targets out of the 102 target genes were found to be connected to at least two compounds, including PTGS2/COX2 (degree = 69), CALM1 (degree = 55), ESR1 (degree = 54), AR (degree = 50), NOS2 (degree = 48), and PPARG (degree = 47), which had higher degrees. These findings provided insight into the related biological functions that participated in the network, where a higher degree indicated a stronger association with AD prevention and treatment.

### Organ-target localization

The target genes screened in this study were used to establish a target-organ localization network with four human organs. The heart (66 targets), liver (65 targets), kidney (10 targets), and brain (34 targets) were identified as the main localization organs (Figure 2C, Supplementary Table 3). These findings suggest that metabolism and the cardiovascular system play vital roles in AD pathogenesis, highlighting the importance of considering other organs besides the brain in the treatment of AD.

### PPI network analysis

**Figure 3:**
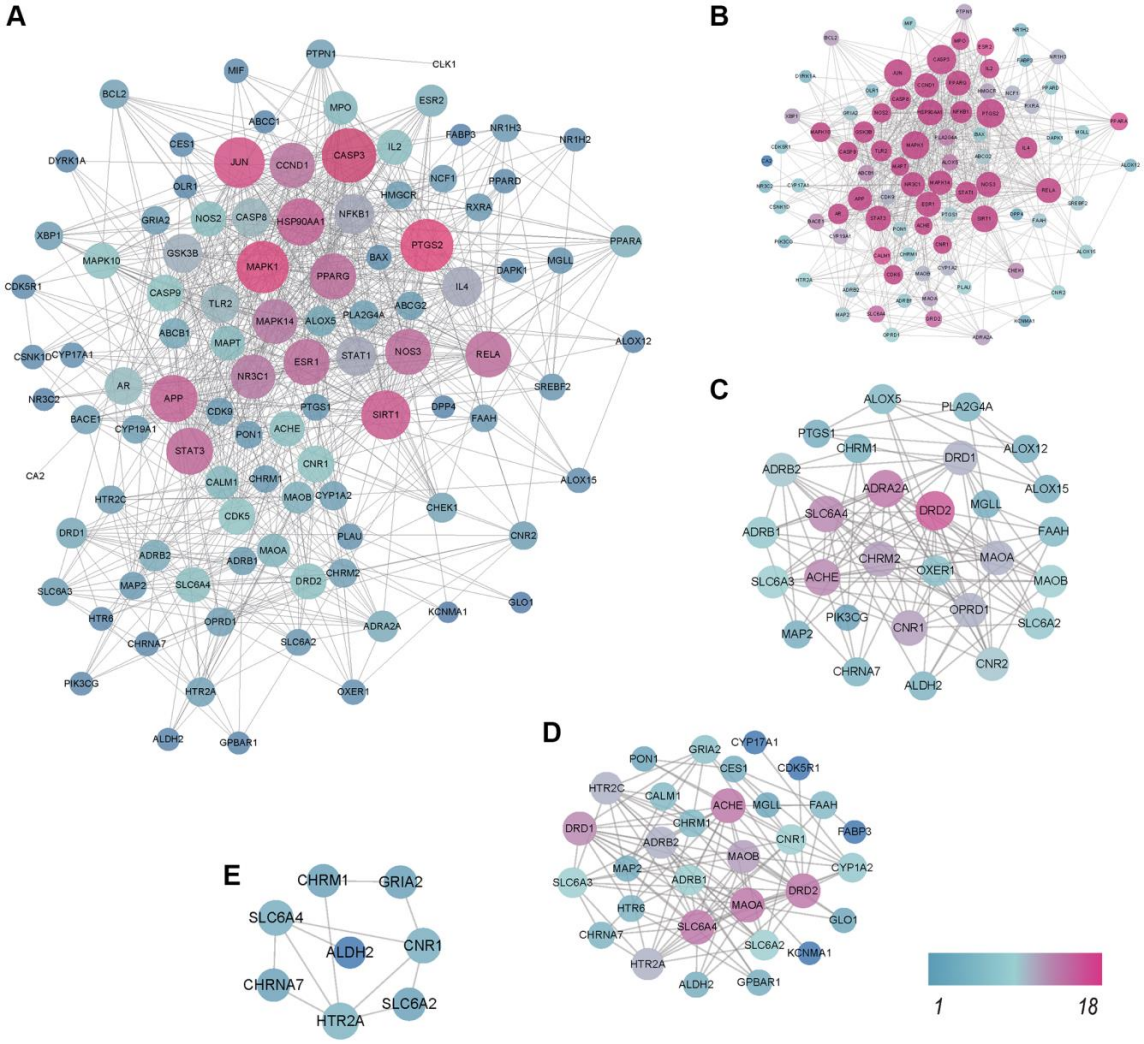
**Protein-protein interaction (PPI) network analysis.** The higher the degree of interaction is, the larger the nodes, and the color changes gradually from blue to red. (**A**) PPI network of 203 proteins and 1876 interaction relationships. (**B**) PPI network of Cluster 1. (**C**) PPI network of Cluster 2. (**D**) PPI network of Cluster 3. (**E**) PPI network of Cluster 4.

To investigate the potential mechanisms responsible for the therapeutic effects of QFY on AD, a PPI network was constructed using the STRING database for a total of 102 potential targets. The resulting network comprised 203 proteins and 1876 interaction relationships (Figure 3A).

Cluster module analysis showed that a total of four cluster modules were obtained from the PPI network (Figure 3B–3E). Cluster 1 was mainly related to metabolic biological processes, including the PI3K-AKT pathway, the AGE-RAGE pathway, and the neurotrophin signaling pathway. Cluster 2 was primarily related to transsynaptic signaling and nervous system development biological processes, as well as neuroactive ligand-receptor interactions, metabolic pathways, and serotonergic synapse pathways. Cluster 3 was related to metabolic processes, transsynaptic signaling biological processes, and neuroactive ligand-receptor interactions as well as dopaminergic synapses, metabolic pathways, and serotonergic synapse pathways. Cluster 4 was related to transsynaptic signaling, chemical synaptic transmission, nervous system processes, and cognition biological processes, as well as neuroactive ligand-receptor interactions, cholinergic synapse, and the serotonergic synapse pathway (Supplementary Table 4). Analysis of the four cluster modules showed that the enriched biological processes and pathways mainly focused on metabolism and synapse pathways.

### GO analysis

**Figure 4:**
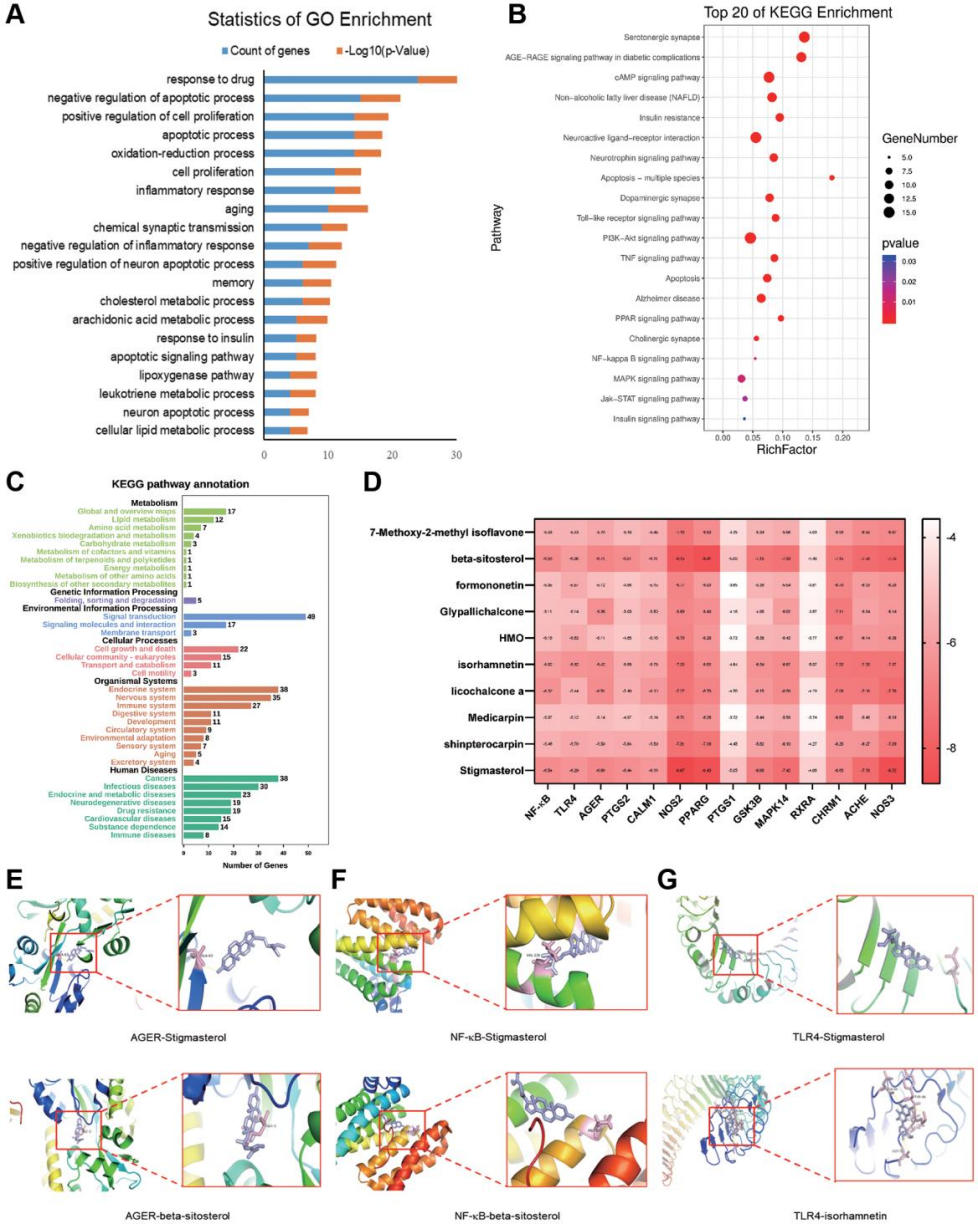
**Gene Ontology (GO) and Kyoto Encyclopedia of Genes and Genomes (KEGG) pathway enrichment analysis and molecular docking of major components to targets.** (**A**) Top 20 biological processes (BP) of GO terms sorted by *P* values < 0.01. Counts of genes and *P* values related to each BP term are shown. The y-axis represents BP terms, and the x-axis shows counts of genes annotated to the BP terms. (**B**) Top 20 enriched pathways. The x-axis shows the number of genes in the given gene set that were annotated to specific pathways, while the y-axis represents the pathways. (**C**) KEGG second class enrichment analysis of 102 potential targets related to AD. The x-axis shows the numbers of genes enriched in certain pathways, while the y-axis represents pathway terms. (**D**) Binding energies of the ligands and receptors. The lower the binding energy is, the greater the stability of the ligand and receptor. (**E**) AGER-stigmasterol and AGER-β-sitosterol. (**F**) NF-κB-stigmasterol and NF-κB-β-sitosterol. (**G**) TLR4-stigmasterol and TLR4-β-isorhamnetin.

To further investigate the potential biological processes involved in QFY’s therapeutic effects on AD, we conducted a GO enrichment analysis on the 102 AD- related targets. The top 20 biological processes were selected and presented in Figure 4A and Supplementary Table 5. The analysis revealed that the pathways involved in QFY’s therapeutic effects on AD were primarily associated with the inflammatory response, metabolic processes, aging, memory, and chemical synaptic transmission.

### Pathway enrichment analysis

We further conducted target-background gene enrichment analysis on 102 AD-related genes and the human genome background genes using the OmicShare (Figure 4C). The signal pathway data enriched by these genes were used to construct a target-signal pathway network map (T-P). There were more targets enriched in metabolic pathways (17 genes), the PI3K-Akt signaling pathway (16 genes), serotonergic synapse (15 genes), and neuroactive ligand-receptor interactions (15 genes) (Figure 4B, Supplementary Table 6). Among these pathways, metabolic pathways, the AGE-RAGE signaling pathway, and serotonergic synapses suggested that QFY mainly alleviates AD by regulating metabolism-related signaling pathways and synaptic activity.

### Molecular docking of the main components of QFY and key targets

The results of molecular docking showed that the potential targets formed stable structures with stigmasterol, β-sitosterol, and isorhamnetin, indicating their potential as effective treatments for Alzheimer’s disease (Figure 4D–4G). The study demonstrated the accuracy of network pharmacology-based predictions and the reasonable selection of core targets and ligands.

### Quality control of QFY freeze-dried powder and drug-containing plasma from rats

**Figure 5:**
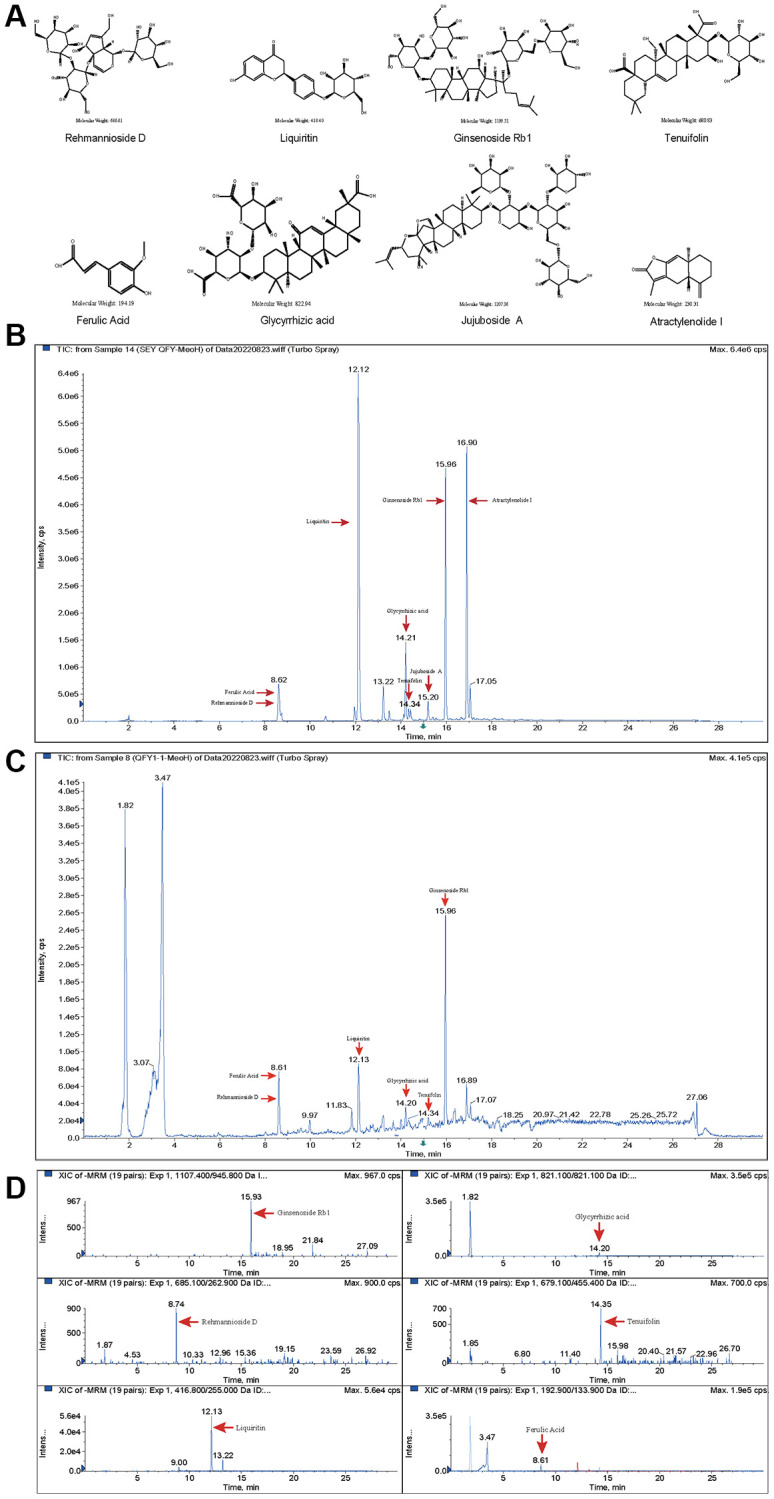
**Component identification of freeze-dried QFY powder and drug-containing plasma from rats.** (**A**) Molecular structure formula of the main compounds of QFY. (**B**) Chromatogram of the main compounds of QFY. (**C**) Chromatogram of the main compounds of QFY-containing plasma. (**D**) Mass spectra of the main compounds of QFY-containing plasma.

Eight quality standards (ginsenoside Rb1, atractylenolide I, rehmannioside D, ferulic acid, jujuboside A, tenuifolin, liquiritin and glycyrrhizic acid) in both freeze-dried QFY powder and drug-containing plasma from rats were identified and quantified by LC-MS/MS. The results showed distinct retention times for each standard, which were 15.93 min, 16.91 min, 8.74 min, 8.61 min, 15.20 min, 14.34 min, 12.12 min, and 14.21 min, respectively (Figure 5A–5C). Furthermore, the quantitative mass spectrometry results revealed the contents of each quality standard in the QFY freeze-dried powder, which were 96.5 mg/kg, 3.2 mg/kg, 84.9 mg/kg, 31.8 mg/kg, 13.8 mg/kg, 13.8 mg/kg, 100.8 mg/kg, and 115.6 mg/kg, respectively. In addition, the concentration of ginsenoside Rb1, rehmannioside D, ferulic acid, tenuifolin, liquiritin, and glycyrrhizic acid in drug-containing plasma were 0.24 μg/ml, 0.16 μg/ml, 0.32 μg/ml, 0.12 μg/ml, 0.04 μg/ml, and 0.08 μg/ml, respectively (Figure 5D). However, atractylenolide I and jujuboside A were not detected in the drug-containing plasma (Figure 5D).

### QFY improves learning and memory ability in AD model rats

**Figure 6:**
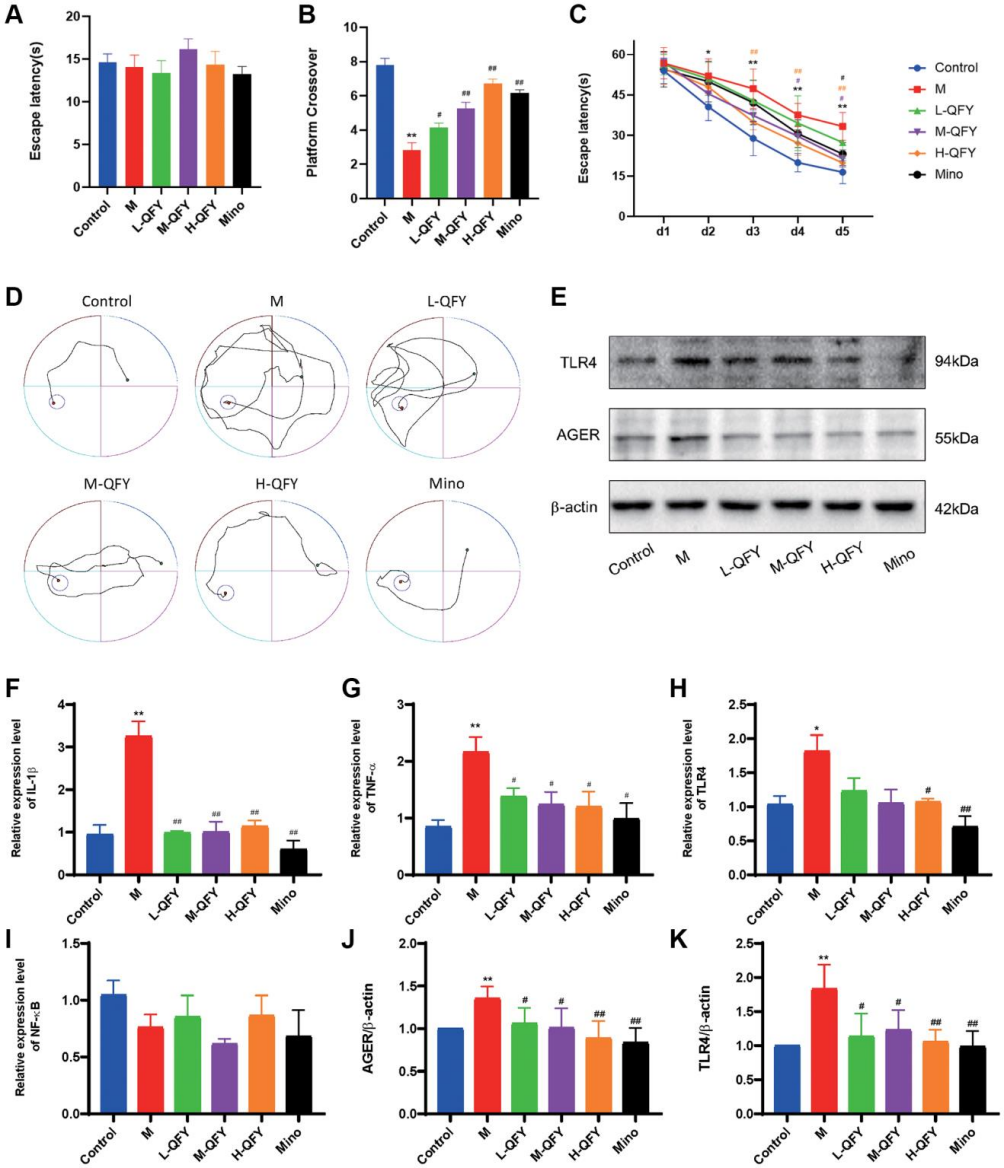
**QFY ameliorates the inflammatory response and improves learning and memory ability in AD rats.** (**A**) Escape latency during the visible platform phase of the Morris water maze test. (**B**) The number of crossings over the previously hidden platform area in the Morris water maze test. (**C**) Escape latency during the acquisition phase of the Morris water maze test. (**D**) The trajectories of the rats looking for hidden platforms on Day 5. *N* = 6 animals/group. The data are expressed as the means ± SDs. ^*^p < 0.05, ^**^p < 0.01, compared with the control; ^#^*P* < 0.05, ^##^*P* < 0.01, compared with the M group; different color symbols represent different groups compared to the model group. (**E**) Detection of TLR4 and AGER protein expression by Western blot. (**F**) The mRNA expression levels of IL-1β in different groups. (**G**) The mRNA expression levels of TNF-α in different groups. (**H**) The mRNA expression levels of TLR4 in different groups. (**I**) The mRNA expression levels of NF-κB in different groups. (**J**) Statistical results of the relative expression levels of TLR4 protein. (**K**) Statistical results of the relative expression levels of AGER protein. *N* = 3 animals/group. The data are expressed as the means ± SDs. ^*^p < 0.05, ^**^p < 0.01, compared with the control; ^#^*P* < 0.05, ^##^*P* < 0.01, compared with the M group.

The MWM paradigm was conducted to investigate the effect of QFY on learning behavior after neuronal damage had already occurred. The control, model, and QFY-treated rats exhibited comparable behavior in the visible platform test. However, the model group displayed reduced platform crossings and longer escape latency compared to the control group. In contrast, the QFY intervention groups exhibited a higher frequency of passing over the previous platform location and a decrease in the avoidance latency, as shown in Figure 6A–6D. These results suggest that QFY may have a beneficial effect on improving learning behavior in mice with neuronal damage in the MWM paradigm.

### QFY downregulates inflammation-related mRNA expression in the brains of AD rats

Effect of QFY on mRNA expression of TLR4, NF-κB and inflammatory factors in the brain of AD model animals by PCR analysis. Our results showed that the expression of TLR4, IL-1β, and TNF-α mRNA was increased in AD model animals, as shown in Figure 6F–6I. However, the expression of TLR4 showed a significant decrease in the H-QFY and Mino groups. Similarly, TNF-α expression was found to be significantly lower in the L-QFY and M-QFY groups. Based on the results, it can be inferred that QFY’s therapeutic effects on AD may be attributed to its ability to modulate the expression of TLR4 and TNF-α. These findings underscore the potential of QFY as a viable treatment option for AD.

### QFY downregulates AGER and TLR4 protein expression in the brains of AD rats

To further investigate the effect of QFY on the protein expression of AGER and TLR4 in the brains of AD model rats, we conducted protein expression analysis. The results revealed that there was a significant increase in the expression of AGER and TLR4 in the AD rats, as demonstrated in Figure 6E, 6J, 6K. However, both QFY and Mino interventions were effective in reversing this elevation. These results suggest that treatment with QFY and Mino may have a suppressive effect on the expression of AGER and TLR4 in the studied model.

### QFY reduces microglial activation and intracellular NF-κB activation in the hippocampus of AD rats

**Figure 7:**
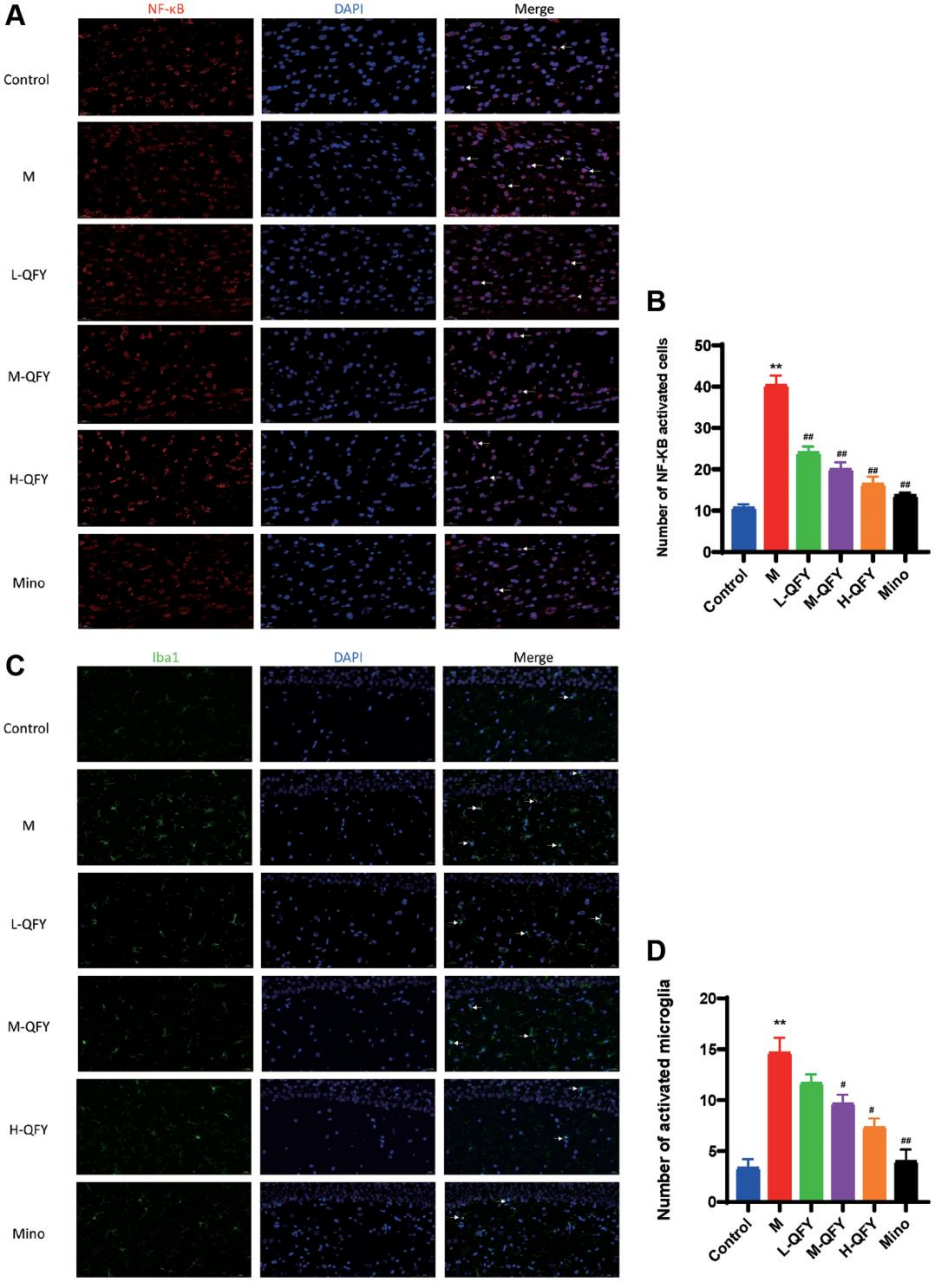
**QFY reduces microglial activation and intracellular NF-κB activation in the hippocampus of AD rats.** (**A**) The expression of NF-κB (red) in the hippocampus of AD rats. Fluorescence (×630). (**B**) Statistical analysis of data for the expression of NF-κB. (**C**) The expression of Iba1 (green) in the hippocampus of AD rats. Fluorescence (×400). (**D**) Statistical analysis of data for the expression of Iba1. *N* = 3 animals/group. The data are expressed as the means ± SDs. ^*^p < 0.05, ^**^p < 0.01, compared with the control; ^#^*P* < 0.05, ^##^*P* < 0.01, compared with the M group.

To further investigate the underlying mechanism of how QFY exhibits its anti-AD activities in the brain, we conducted an immunofluorescence analysis of Iba1 and NF-κB. Our results, as shown in Figure 7, demonstrated higher levels of NF-κB in the nucleus and Iba1 in the model group compared to the normal group. However, both QFY and minocycline were effective in reducing NF-κB expression in the nucleus and microglia activation in the hippocampal region of AD model rats. The results indicate that QFY may have a beneficial impact on AD by reducing microglia activation and NF-κB expression, further supporting its potential as a treatment option for AD.

## DISCUSSION

In this study, we utilized network pharmacology analysis to elucidate the mechanisms underlying the therapeutic effect of QFY in AD. We first identified the active ingredients and predicted targets of QFY, followed by disease correlation analysis and visualization of the C-T and C-T-AD networks. Subsequently, we performed a PPI network analysis to further explore the potential targets of QFY in AD and verified by molecular docking. Simultaneously, a target-organ localization network between the screened targets and 84 organs in the human body was established to investigate the potential organ targets of QFY. Further, GO and pathway enrichment analyses were constructed to reveal the action pathway of QFY in treating AD. Finally, an animal experiment was carried out to verify the results of bioinformatics. Based on these studies, we explored the mechanisms of QFY in treating AD.

### Principal findings and comparison with other studies

Our results revealed a total of 203 potential targets among 87 active compounds in QFY, with each compound having approximately 45 targets. The results demonstrate that the treatment effect of QFY on AD is likely due to its combined effect on multiple targets, highlighting its potential as a possible therapeutic strategy for treating AD. In our network pharmacology analysis, we identified 102 potential targets related to AD from among 87 compounds in QFY. Notably, isorhamnetin, formononetin, 7-methoxy-2-methyl isoflavone, stigmasterol, and β-sitosterol were among the main active compounds in QFY, with a higher number of predicted targets. This suggests that these compounds may play a crucial role in the pharmacological action of QFY in the treatment of AD. Isorhamnetin alleviates inflammatory response and apoptosis through the Akt/SIRT1/Nrf2/HO-1 signaling pathway [35], regulates apoptosis and autophagy through the P38/PPAR-α pathway [36], and protects against Aβ-induced cytotoxicity and Aβ aggregation [37]. Formononetin has been found to have potential benefits in protecting against AD and regulating vascular inflammation in AD through the Nrf2 pathways [38]. It also has a neuroprotective effect and can improve learning and memory in a mouse model of AD [39]. Further, it has identified seven key target genes for formononetin in the treatment of patients with AD. The biological processes included apoptosis, energy pathways, metabolism, and signal transduction [40]. β-Sitosterol also has potential treatment effects on the management of AD. For example, β-sitosterol gradually improved the working memory, spontaneous alternation behavior, and motor coordination of transgenic mice [41]. β-Sitosterol is beneficial in neurodegenerative diseases such as AD by promoting inner mitochondrial membrane fluidity to enhance mitochondrial function [42] and alleviating inflammation via ERK/p38 and NF-κB pathways [43]. Stigmasterol can decrease the phosphorylation of MAPKs to protect neurons against Aβ_25-35_-induced injury, with anti-AD effects [44]. According to the results of molecular docking analysis, stigmasterol formed stable structures with NOS2, PPARG, MAPK14, AGER, and NOS3, while β-sitosterol showed better binding activity and stable conformations with PTGS1, PTGS2, GSK3B, ACHE, NF-κB, and other targets. These findings suggest that the active ingredients in QFY may improve cognitive function in AD by modulating inflammation-related targets.

A target-organ localization network between the screened targets and 84 organs in the human body was established to investigate the potential organ targets of QFY. The results showed that the lungs, heart, liver, kidneys, brain, and blood were the main organs associated with the potential drug targets of QFY. Interestingly, the targets of QFY are located highly not only in the brain but also in other organs, especially the liver and blood, which may be related to drug absorption, metabolism, and clearance. Therefore, it is necessary to consider the comprehensive effects of QFY on multiple organs and systems when developing therapeutic strategies for the treatment of AD.

The results of GO and pathway analyses revealed that the action pathway of QFY in treating AD was primarily related to metabolism, inflammatory response, apoptosis, aging, and memory. The alterations in energy metabolism in normal brain aging and AD, including glycolipid metabolism and mitochondrial metabolism, were connected to inflammatory responses via redox regulation [45]. Inflammatory response and apoptosis are also important processes that contribute to neuroinflammation and neuronal cell death in AD. Aging is a primary AD risk factor, and memory impairment is a hallmark symptom of AD. Similarly, NF-κB, STAT3, MAPK, PPARG, caspase 3 (CASP3), and NOS in the C-T-AD network showed higher enrichment. These findings indicated that these genes are the key part of the network and that inflammation, apoptosis, and metabolism may be the main processes through which QFY treats AD. Therefore, the regulation of these biological processes and signaling pathways may be key mechanisms underlying the therapeutic effects of QFY in the treatment of AD.

The enrichment analysis revealed that the AGE-RAGE pathway was significantly enriched. The binding of AGEs to their receptor RAGE activates a range of signaling pathways, including PI3K-AKT, JAK-STAT, MAPK, and NF-κB signaling. The accumulation of AGEs is a hallmark of connective tissue aging and is characterized by a gradual decline in regenerative capacity [46]. AGEs can upregulate the mRNA expression of MCP-1, TNF-α, IL-6, IL-1, COX-2, and iNOS and activate the RAGE/NF-κB pathway [47]. The prolonged cellular disturbance brought on by ligand-RAGE interactions is a hallmark of chronic diseases such as diabetes, inflammation, and AD. As a pattern recognition receptor, overexpression of RAGE can trigger a range of signals, including PI3K/Akt and MAPK pathways [48]. These signals ultimately lead to NF-κB activation and inflammation [49]. Besides, RAGE enables extracellular HMGB1-LPS complex transport to lysosomes to activate cytosolic caspase-11 in macrophages and endothelial cells [50]. Abnormal activation of JNK occurs in AD patients and AD transgenic mice, and inhibition of JNK activation can reduce neuroinflammation and synaptic loss [51]. JNK is a subfamily of MAPKs and plays a critical role in the regulation of insulin signaling, inflammation, apoptosis, and caspase-3 activity in diabetes and the upregulation of pro-inflammatory cytokines such as MCP-1, IL-6, IL-8, and TNF-α in AD pathology [52]. ERK and p38 MAPK, the two other main members of the MAPK family, also play a role by working together to activate NF-κB signaling, leading to neuroinflammation [53]. Moreover, the activation of the JNK and p38 MAPK pathways can promote apoptosis [54]. Apoptosis is an important mechanism leading to synaptic dysfunction and neuronal loss in AD [55]. The results of a genome-wide association study (GWAS) suggested that abnormalities in JAK-STAT signaling were associated with the pathogenesis of AD [56]. Moreover, the levels of STAT3 are reduced in the hippocampus of AD patients [57]. The activation of STAT3 has been shown to improve cognitive deficits in animal models of AD through the regulation of NMDA receptor (NMDAR) expression [58]. In summary, we predicted that QFY can alleviate neuroinflammation and apoptosis through RAGE-related pathways.

Proteins collaborate within the PPI network to carry out essential molecular processes within the cell. Anomalies in a specific protein can disrupt the function of other proteins in the network, ultimately leading to the development of a disease. Consistent with the results of enrichment analysis, the PPI network between QFY and AD displayed significant interactions with NF-κB, STAT3, MAPKs, and TLRs. The primary enrichment pathways identified in Cluster 1 were the AGE-RAGE pathway and the PI3K-AKT pathway. The NF-κB is particularly notorious for its role in mediating inflammatory responses and its canonical pathway is a key characteristic in AD development [59]. TLRs are transmembrane proteins that initiate innate and adaptive immune responses through recognized cellular damage-associated molecular patterns (DAMPs). However, dysfunctional or excessive TLR activation can contribute to various dysfunctions such as autoimmune, inflammatory, and age-associated diseases [60]. TLR4 activation leads to NF-κB activation and proinflammatory cytokine release through the activation of both myeloid differentiation primary response protein 88 (MyD88)-dependent and MyD88-independent signals [61]. Based on our analysis, we hypothesize that the mechanism underlying the therapeutic effects of QFY on AD involves the RAGE pathway and NF-κB pathway, including targets such as MAPKs, TLRs, and NF-κB. Our study suggests that QFY exerts its beneficial effects by inhibiting neuroinflammation, which is a key contributor to the development and progression of AD.

During our research, network pharmacology analysis indicated that the anti-AD mechanism of QFY is associated with inflammation, and this mechanism may be mediated by AGER and NF-κB. Minocycline, a broad-spectrum tetracycline antibiotic, is a microglial cell inhibitor that can suppress microglia activation and reduce the expression and release of inflammatory mediators [62, 63]. Through its anti-inflammatory and neuroprotective actions, minocycline has been shown to have a beneficial effect in ameliorating spatial memory decline [64]. As a result, we chose minocycline as our experimental positive control drug. Our MWM test results demonstrated that QFY can partially enhance spatial memory. Both TLR4 and AGER are capable of activating the downstream target NF-κB, thereby promoting inflammation upon binding to their respective ligands [65]. Extracellular AGEs can directly bind to myeloid differentiation 2 (MD2), which is a co-receptor of TLR4. This binding leads to the formation of an AGEs-MD2-TLR4 complex and initiates pro-inflammatory pathways [66]. Meanwhile, RAGE has been discovered in a variety of immune cells that are essential for maintaining the immune response. It has been found that many of the extracellular ligands that initiate RAGE signaling are implicated in both acute and chronic immune responses. Following RAGE activation, the proinflammatory transcription factor NF-κB and its downstream target genes are induced. Interestingly, RAGE has a functional NF-κB binding site in its proximal promoter, making it an NF-κB-regulated target gene as well [67]. Our findings are consistent with the hypothesis that QFY can ameliorate the inflammation of the brain by reducing the protein expression of TLR4 and AGER. Specifically, QFY intervention reversed the upregulation of TLR4 and AGER protein expression in AD rats. Additionally, our immunofluorescence results revealed that QFY reduced the entry of NF-κB into the nucleus, thereby inhibiting NF-κB activation. Although there were no significant differences in the NF-κB mRNA expression among the different groups, this can be attributed to the fact that mRNA represents the total expression of NF-κB. The entry of NF-κB into the nucleus represents its activation, which in turn promotes the activation of microglia. Therefore, it is normal that there is a difference between the total expression and activation of NF-κB. Furthermore, QFY reduced the mRNA expression levels of inflammatory factors such as IL-1β and TNF-α in AD rats. Additionally, QFY reduced microglia activation, as microglia are the main mediators of neuroinflammation and the natural immune cells of the central nervous system. Our results suggest that QFY can exert anti-inflammatory effects in the brain through multiple pathways, thereby enhancing learning and memory in AD rats.

### Strengths and limitations of the study

This study not only predicted the target of QFY in treating AD but also validated the main predicted targets and pathways through animal experimentation. The main target sites and mechanisms of QFY in the treatment of AD have been identified, which is mainly achieved through the inhibition of inflammatory activation of microglia in the brain via the RAGE and TLR4/NF-κB signaling pathways. This leads to the improvement of neuroinflammation and learning and memory abilities in the AD model rats. These findings provide scientific evidence for the clinical application of QFY in the treatment of AD.

Our study has some limitations. Firstly, the study relied on network pharmacology and molecular docking analyses, which were subsequently validated through animal experimentation. However, the specific molecular mechanisms underlying the therapeutic effects of QFY in treating AD remain limited. We were unable to identify the main active ingredients within QFY responsible for its therapeutic effects on AD, particularly concerning the RAGE and TLR4/NF-κB signaling pathways. Although our molecular analyses revealed that Stigmasterol and β-sitosterol present in QFY could form stable structures with AD targets, these results lack comprehensive experimental verification. This limitation can be attributed to our primary focus on the overall therapeutic function of QFY in treating AD. Traditional Chinese Medicine often employs a prescription approach to treat diseases, wherein the mechanism of action is multifaceted, encompassing not only the individual components but also the interactions and metabolism of the prescription as a whole.

## CONCLUSIONS

In summary, our systematic pharmacological analysis identified the potential targets of QFY for the treatment of AD. Our findings suggest that QFY can reduce neuroinflammation by inhibiting both RAGE and TLR4/NF-κB pathways and microglia activation in AD model rats, leading to improved learning and memory in these animals. Further research is required to validate the predicted targets of QFY and to develop a more comprehensive understanding of its efficacy and mechanism in treating AD.

## Supplementary Materials

Supplementary Figure 1

Supplementary Table 1

Supplementary Table 2

Supplementary Table 3

Supplementary Table 4

Supplementary Table 5

Supplementary Table 6
