# Supplementary material for: Qi Fu Yin ameliorates neuroinflammation through inhibiting RAGE and TLR4/NF-κB pathway in AD model rats
Source: Aging (Albany NY). 2023 Nov 22;15(22):13239–64. doi: 10.18632/aging.205238 (PMC10713403; doi:10.18632/aging.205238)
Supplement: Supplementary Figure 1 [file aging-15-205238-s001.pdf]

SUPPLEMENTARY FIGURE

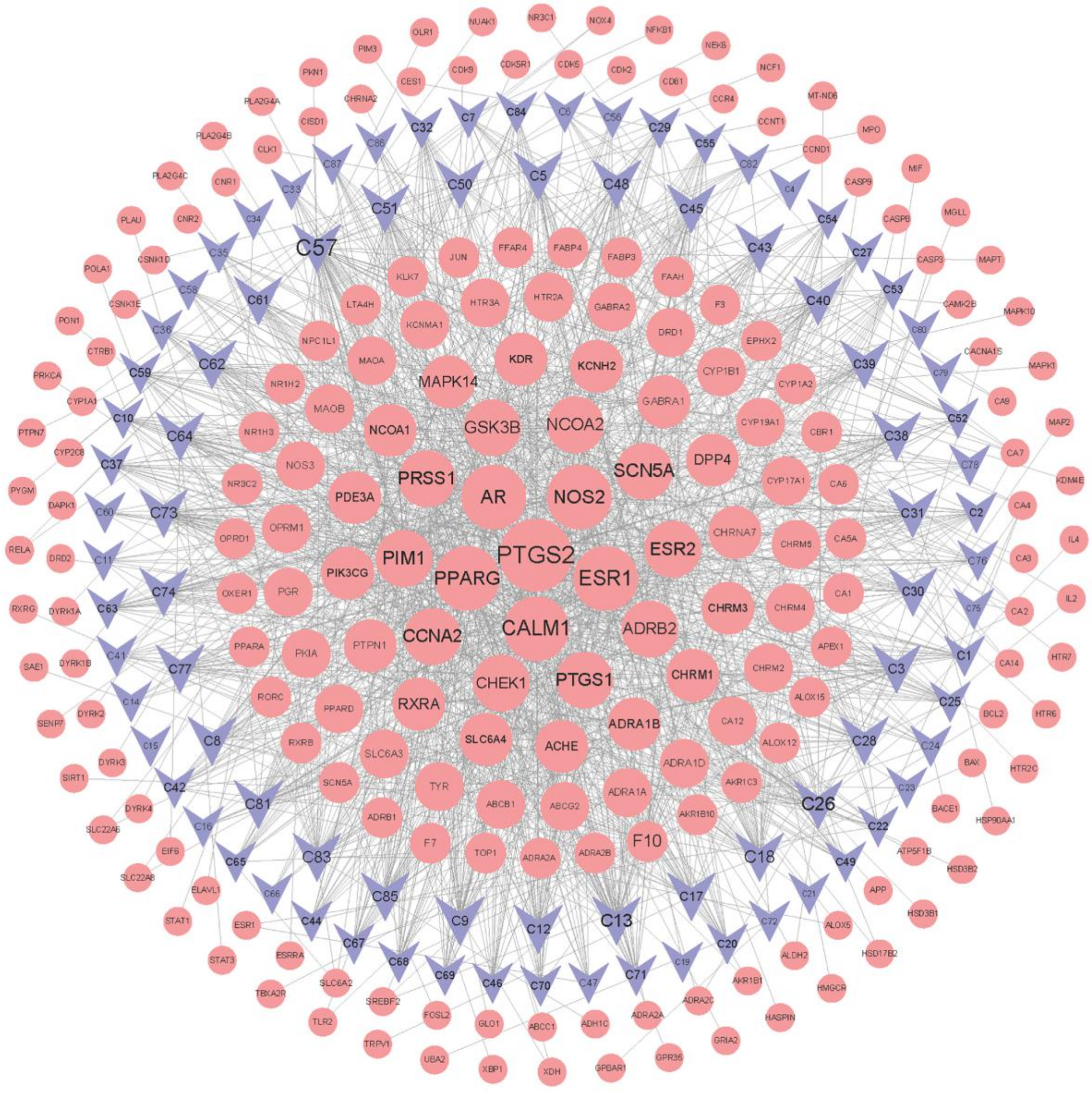

Supplementary Figure 1. C-T network based on the interactions between 87 compounds and 203 targets in QFY. The purple nodes represent compounds, the pink nodes represent targets, and the lines between the nodes represent the interactions between compounds and targets.
